# Evaluation of different surgical approaches to remove dental implants from the maxillary sinus

**DOI:** 10.1038/s41598-021-83721-z

**Published:** 2021-02-24

**Authors:** Zaid Hamdoon, Nazhat Mahmood, Wael Talaat, Abier Abdul Sattar, Karrar Naeim, Ahmed Qais, Waad Kheder, Waseem Jerjes

**Affiliations:** 1 College of Dental Medicine, Oral and Craniofacial Health Sciences, University of Sharjah, Sharjah, United Arab Emirates; 2 Almustansyria University-College of Dentistry, Baghdad, Iraq; 3 Iraqi Ministry of Health, Baghdad, Iraq; 4 University of Mosul-College of Dentistry, Mosul, Iraq; 5 North End Medical Centre, London, UK; 6 Suez Canal University, Ismailia, Egypt

**Keywords:** Medical research, Risk factors

## Abstract

Dental implant surgery on atrophied maxilla has many risks; in some patients, simultaneous sinus lifting with implant placement must be performed to increase the chances of successful implantation; this procedure can cause implant migration**.** Eleven patients were diagnosed with implant migration into the maxillary sinus in four anatomical areas: the sinus floor above the alveolar bone, near the junction of the sinus and nasal floor, near the floor of the orbit, and the most posterior aspect of the sinus. Surgical removal was performed through four different direct non-endoscopic transoral approaches depending on the location of the displaced implant. Surgical challenges, surgery duration and postoperative complications were reported. The least challenging surgical intervention was noted when removing the displaced implants from the floor of the sinus through the crestal approach. More challenges were experienced during the surgical removal of anteriorly displaced implants near the roof of the orbital floor due to surgical access and the proximity of vital anatomical structures. Bleeding from the pterygoid venous plexus was profound with the posterior lateral approach. The choice of an appropriate surgical approach to retrieve displaced dental implants from the maxillary sinus depends on the location of the implant and the surrounding vital anatomical structures.

## Introduction

Poor bone quality and low quantity as well as proximity to vital anatomical structures, e.g. maxillary sinus, leads to surgery of the posterior maxilla being associated with a high level of complications. Soft bone and/or overpreparation of the implant site may result in primary instability and increase the risk of sinus perforation with displacement of the dental implant into the sinus^1,2^.

The implant may become displaced and enter the sinus during surgery due to insufficient primary stability of the implant during implantation or due to bone resorption around the implant in the following months postoperatively. This can lead to implant migration towards the maxillary sinus due to the forces created by the mechanism of mastication. The critical time is usually during the first three postoperative weeks when the bone is undergoing remodelling; hence, implant stability may be compromised^3^.

Insertion of dental implants without a sinus lifting procedure in highly pneumatized sinuses can lead to implant migration into the sinus floor. The existence of untreated antral base perforation following alveolar sinus lifting facilitates more distant implant displacement. Such displacement might not be limited to the sinus floor but may include other locations in the sinus. In some cases, the displacement may not be limited to the maxillary sinus or other structures, including the paranasal sinuses. Such diversity in the displacement locations warrants the use of different surgical approaches to retrieve these implants^4–6^.

Different endoscopic and non-endoscopic sinus surgeries have been reported over the years to remove displaced implants from the maxillary sinus^7–9^. However, to our knowledge, no study has yet investigated the efficacy of different intraoral approaches. In this case series, four anatomic forms of displacement were discussed, and modified and non-modified Caldwell-Luc surgical approaches were performed to retrieve these implants. The aim of the present study was to evaluate the advantages and disadvantages of different surgical approaches used to retrieve migrating dental implants from inside the maxillary sinus.

## Patients and methods

This prospective clinical series involved 11 patients who presented to the Oral and Maxillofacial Unit, Yarmouk Dental University College, IRAQ with displaced dental implants inside the maxillary sinus and other associated symptoms from February 2017 to March 2019. The study was approved by the Yarmouk Dental University College Human Ethics Committee and was carried out in accordance with the Declaration of Helsinki. All patients signed an informed consent form to receive treatment and to be included in this study. Implant migration was identified in 5 male and 3 female patients, with an age range of 35–56 years. Our data showed that two patients presented within the first 24 h of displacement, 8 patients presented within the first 8 weeks of implant placement, and one presented 6 months after loading. All the patients exhibited unremarkable medical histories. Five of the patients were chronic smokers, and two drank alcohol socially (non-chronic). The group demographics are highlighted in Table 1.

**Table 1:** Demographic information.

Variable	Gender	Age	Smoking	Alcohol	ASA	Location	Sinus lifting
Patient 1	Male	38	Yes	No	I	1st PM	By implant*
Patient 2	Male	55	No	Yes	II	2nd PM	By implant
Patient 3	Male	45	Yes	No	I	1st PM	By implant
Patient 4	Male	51	Yes	No	I	2nd PM	Crestal ≠
Patient 5	Male	53	No	Yes	I	1st M	Crestal
Patient 6	Male	56	Yes	No	II	2nd PM	Crestal
Patient 7	Female	35	No	No	I	1st M	Crestal
Patient 8	Female	39	Yes	No	II	2nd M	Crestal
Patient 9	Female	41	No	No	I	1st M	Crestal
Patient 10	Female	39	No	No	I	1st M	Crestal
Patient 11	Female	40	No	No	I	1st M	Crestal

By implant*: sinus lifting by the implant’s apex without bone graft.

Crestal ≠ : crestal sinus lifting using the osetotome and bone graft augmentation.

Prior to their presentation, eight patients underwent crestal sinus lifting using an osteotome technique with simultaneous bone graft and implant placements, and the other three underwent sinus lifting by the implant without bone graft. All the cases, except one, were referred to our unit. An OPG (orthopantomogram) with CBCT (cone beam computed tomography) was used to investigate the anatomical location of the displaced dental implant.

**Figure 1:**
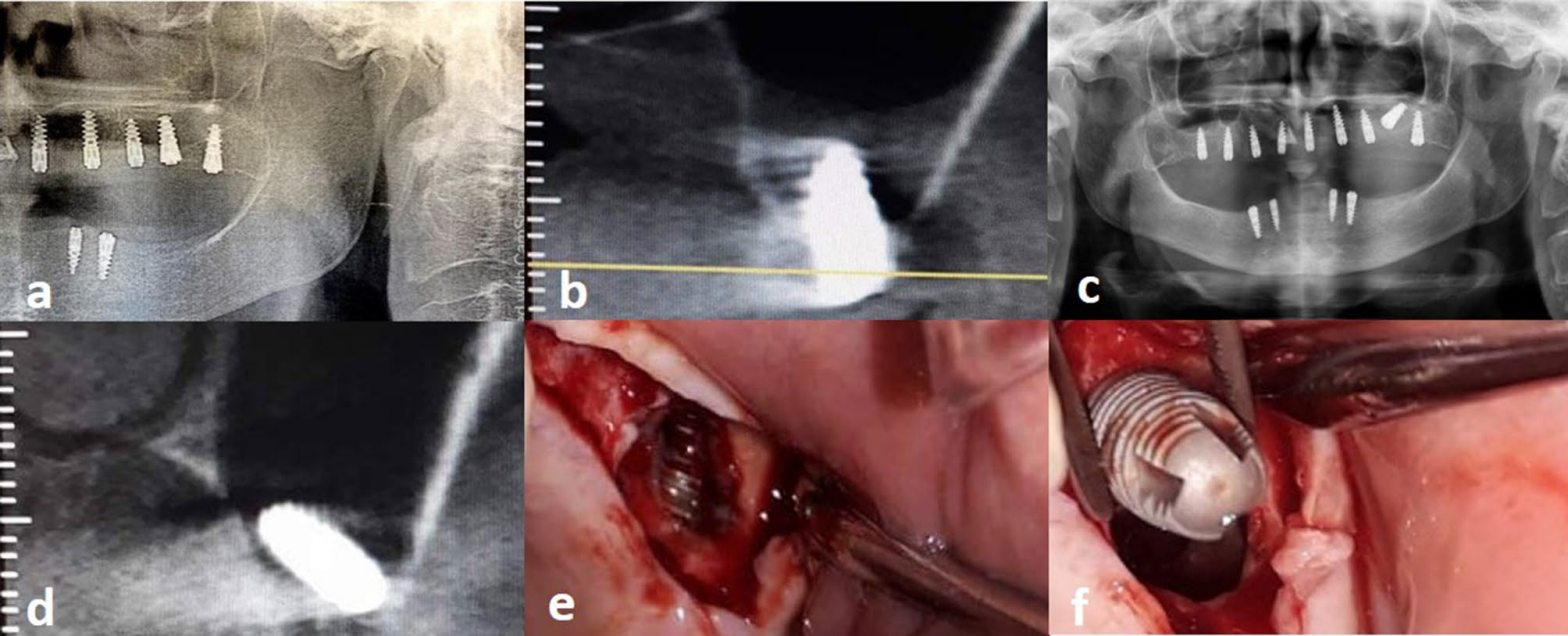
Two dimentional  X-ray exhibiting a limited vertical residual bone in the upper posterior maxillary area (**a**); immediate coronal CBCT showing implant surrounded by bone graft (**b**); implant migration three weeks after crestal sinus lifting (**c**,**d**); envelope crestal incision, surgical window in the alveolar bone followed by implant catching and retrieval through transalvoelar approach (**e**,**f**).

**Figure 2:**
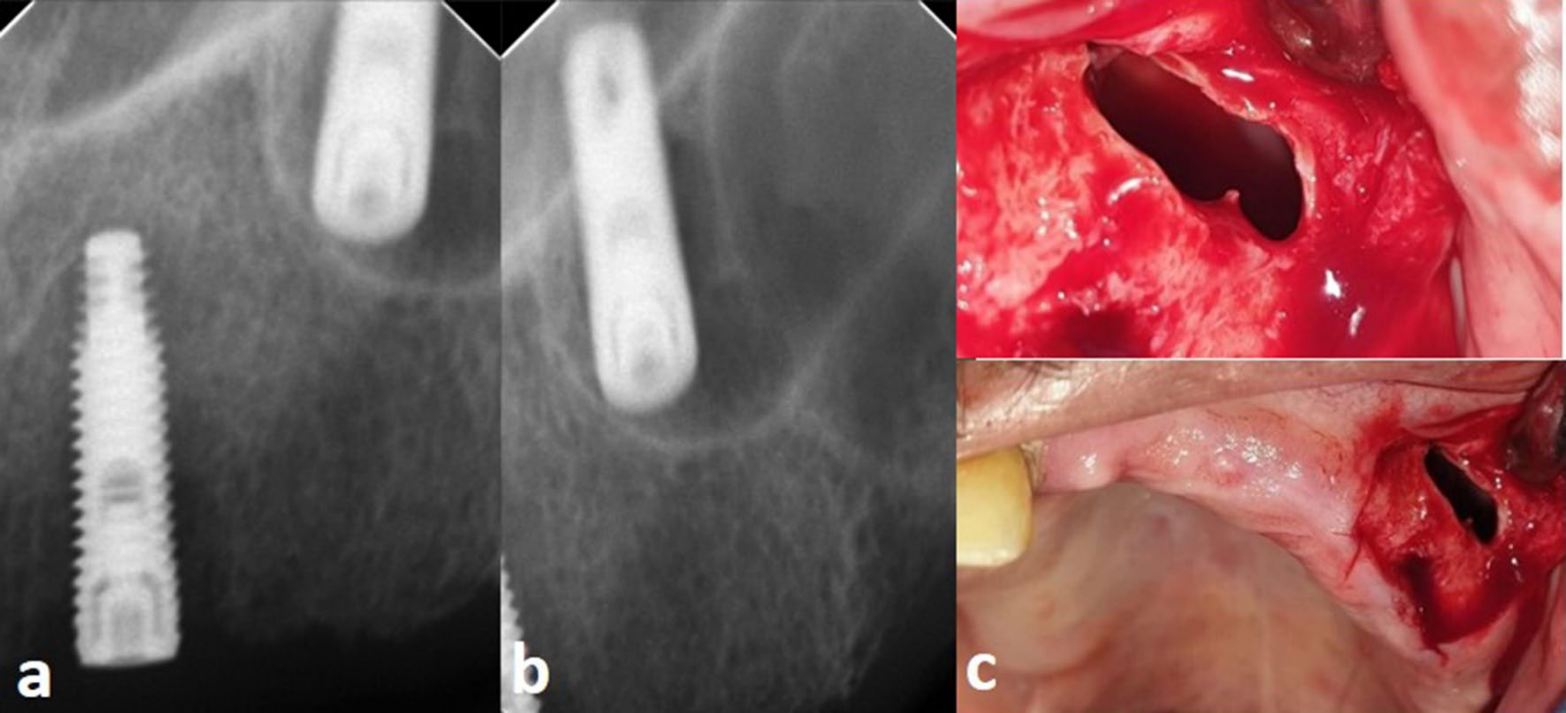
Dental implant displacement toward the most anterior part of the sinus and near to the nasal cavity (**a**,**b**); Caldwell Luc operation near the canine fossa with three sided flap and wide bone window to retrieve the implant (**c**).

**Figure 3:**
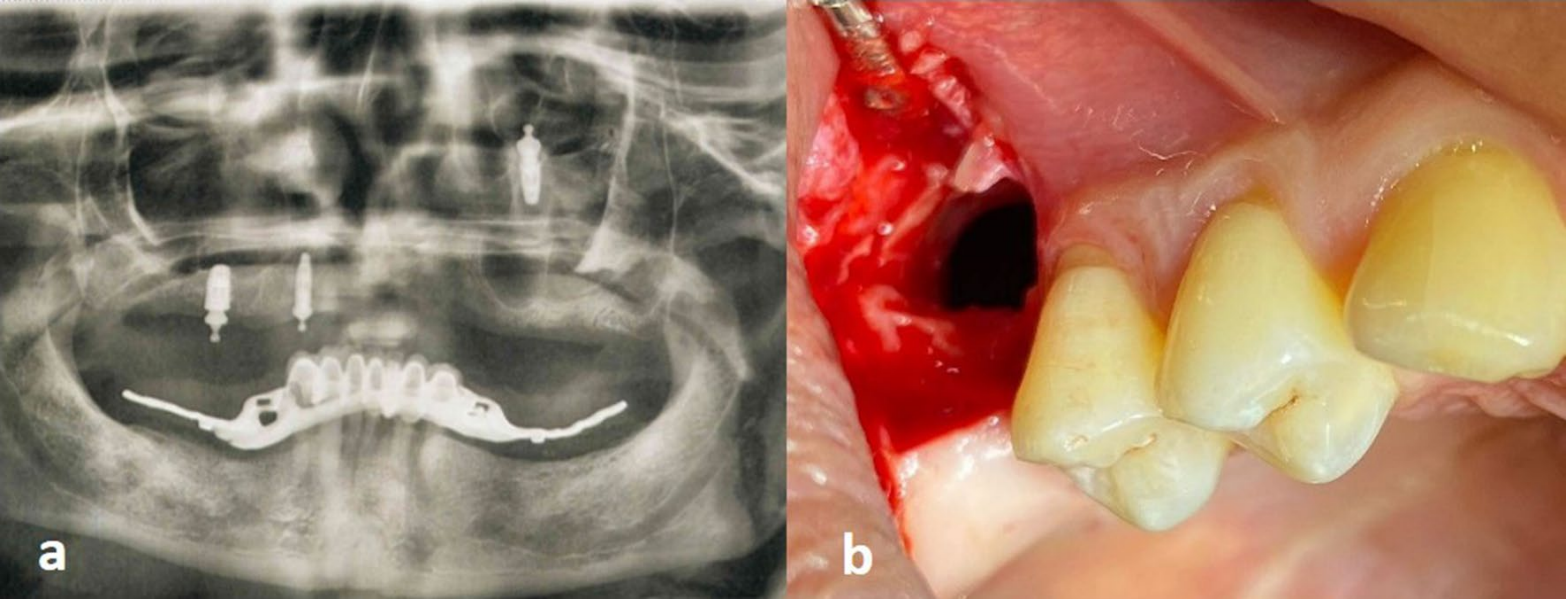
An X-ray showing a dental implant with ball abutment displaced toward the superior part of the maxillary sinus 6 months after function (**a**); upper lateral surgical approach to access displaced implant in the superior part of the sinus using two sided flap (**b**).

**Figure 4:**
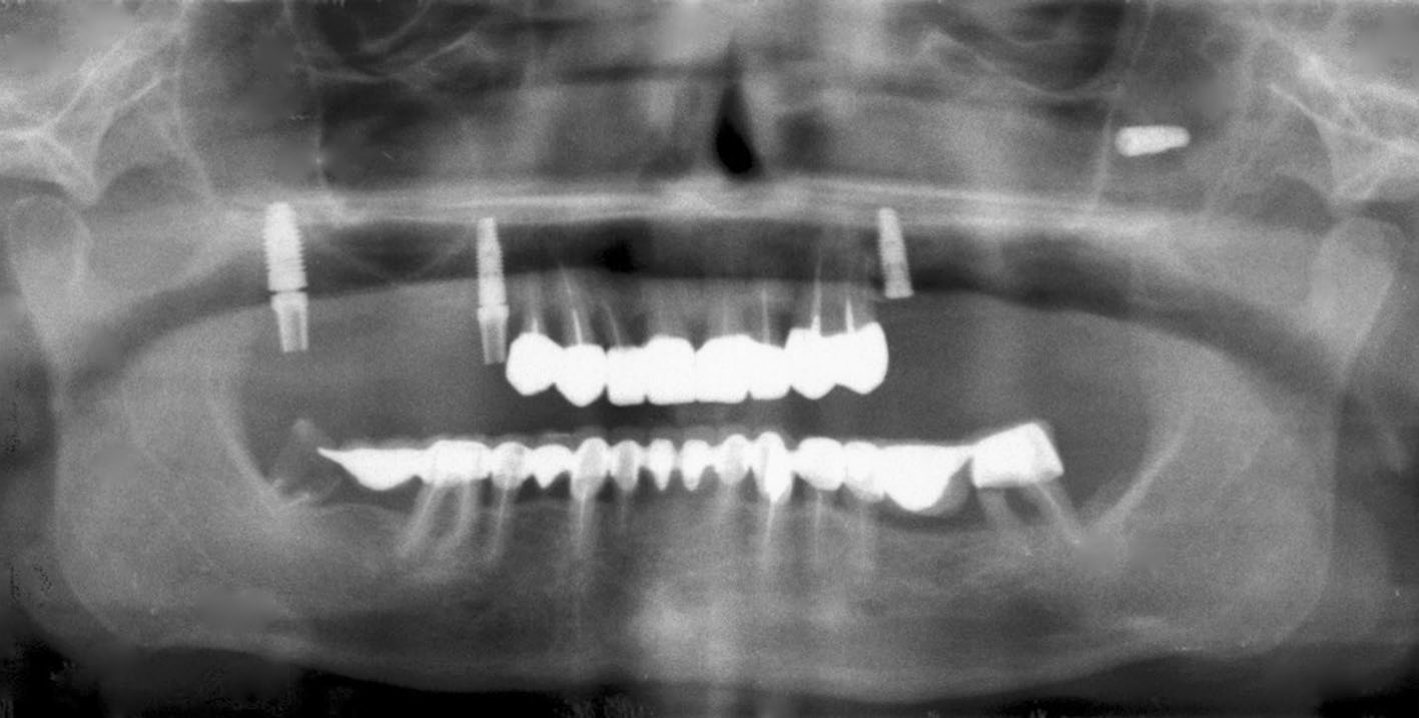
Implant displacement in the most posterior part of the sinus.

**Figure 5:**
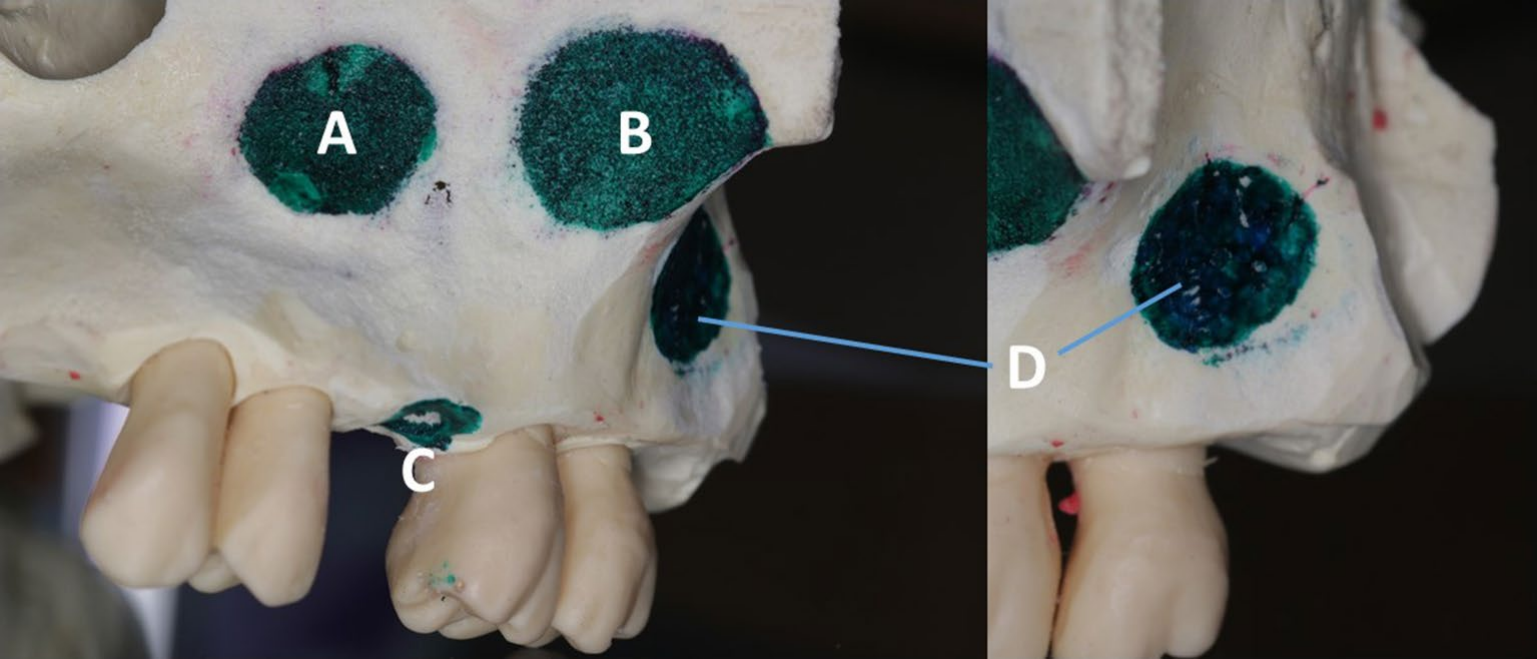
A surgical model representing different trans-oral surgical approach to remove dental implant from the maxillary sinus. Caldwell-Luc anterior approach (**A**); upper lateral approach (ULA) under the zygomatic (**B**); intracrestal (lower window) approach through the implant insertion site (**C**); posterior lateral approach (**D**).

Four main classical approaches (non-endoscopic direct intra-oral) were used for implant retrieval depending on its location: an intracrestal (lower window) approach through the implant insertion site (Fig. 1), a Caldwell-Luc upper procedure (anterior approach) through the bony window near the canine fossa (Fig. 2), an upper lateral approach (ULA) under the zygomatic buttress for a displaced implant in the superior aspect of the sinus (Fig. 3) and a posterior lateral approach (PLA) for a displaced implant in the posterior aspect of the sinus (Fig. 4). In the latter approach, the posterior part of the lateral wall of the maxillary sinus was breached by making a window by Christmas tree round bur near and above the maxillary tuberosity (Fig. 5).

Antimicrobial prophylaxis was administered (amoxicillin 1 gm BD for 5/7, with the first dose to be taken 1 h preoperatively). The decision for the surgical approach depended mainly on the surgeon’s experience, the anatomical location of the displaced implant and the availability of a surgical endoscope in our unit. Local anaesthesia by infiltration with lidocaine/epinephrine was carried out. Then, a crestal incision was made before a full thickness mucoperiosteal flap was raised, with the aim of exposing the anterior-lateral wall of the maxilla from the canine to the molar region for the Caldwell-Luc approach (CLA). Intra-oral X-rays were used during the operation to define the anterior boundary of the maxillary sinus and to differentiate between the maxillary sinus and nasal cavity.

The upper lateral antrostomy was carried out at the premolar-molar region, the flap extended more superiorly, bone was removed, and the sinus membrane was raised and incised to allow removal of the dental implant. The membrane was sutured with a 6–0 Vicryl suture. A resorbable membrane was used to protect the elevated sinus membrane. A plastic surgical aspirator tip was fitted tightly into the access window in case of far displaced implants. The sinus was filled with saline, and the patient was placed in a lateral position on the dental chair with the sinus involved in surgery positioned on the underside. Saline served as a medium to bring out the implant.

All surgeries were carried out by a single experienced operator who was later asked to rate the surgical complexity of each approach as easy, moderate, difficult or highly difficult and include an explanation. The time required to gain access and remove the implants and the early and late postoperative complications were recorded. Nasal obstructions were evaluated at day 7 from the surgery by asking the patient to close the nostril on the side of the surgery and breathe in and out normally from the normal nostril after placing cotton wool 1 cm from the nostril; the patients were then asked to breathe in and out of the nostril on the surgery side with the normal nostril closed. According to the movement of the wool, the obstruction was categorized relative to the normal side into normal nasal flow, mild obstruction with less vibration of the fibres, moderate obstruction with limited movement of the wool and severe obstruction in which no movement of the fibres was observed.

Facial and infraorbital nerve conduction were tested at 14 days, 1 month and 3 months after the surgery with a two-point discrimination test and cotton wick test on the buccal gingiva and skin over the zygomatic bone, nose and upper lip. Facial swelling was measured at day 7 using measuring tape from the ala of the nose to the tragus of the ear of the normal side and compared with the surgery side. An increase in the measurement of less than 1 cm was considered mild swelling, an increase of 1–2 cm was considered moderate swelling, and swelling of more than 3 cm was considered severe swelling.

A questionnaire was given to each patient at the end of surgery that included three elements: pain, taste changes and post-nasal drip evaluation. The visual analogue scale (VAS) was used to evaluate the severity of the pain from 1 to 7 days post-operatively and as reported by the patient. The VAS ranged from 0 (no pain) to 10 (terrible pain), a score of 1 to 3 was indicative of mild pain, 4 to 6 was indicative of moderate pain, and 7 to 10 was indicative of severe pain. A foul taste was evaluated subjectively at day 14 according to the duration and severity of the altered taste. Insignificant foul taste was defined in the questionnaire as an unpleasant taste present in early morning that disappeared in the afternoon, and experiencing an abnormal taste for a full day that increased in the morning and deceased in the evening was rated as significant taste alteration. Highly significant altered taste was defined as a rotten taste that was experienced continuously during the day and night. In the post nasal drip, the patients were asked at day 7 if they experienced any excess mucus in the back of their nose dripped down the back of the throat. In the questionnaire, the patient needed to answer whether the drip existed without further explanations.

In addition to the questionnaire, all the patients were called for a review appointment at 7, 10 and 14 days post-operative to check wound status and to confirm that the ratings in the questionnaire were understood and written properly. Post-operative follow-ups were scheduled at weekly intervals from 14 days up to 3 months post-operatively to monitor the healing process, record outcomes and manage any complications. Sutures were removed 2 weeks following surgery. Patients were recommended to consume a soft diet for 4 weeks, and oral hygiene instructions were provided. Non-steroidal anti-inflammatory drugs (Ibuprofen 600 mg TDS for 5 to 7 days) and nasal decongestant (Pseudoephedrine nasal drop, BD for 3 to 7 days) were issued to each patient.

### Ethics

Ethical approval has been obtained from Yarmouk Dental University College (File: 382K-2018). All the patients in this study provided informed consent.

## Results

The average time required to remove the implants from the sinus using the low window (intracrestal approach) was 11.5 min compared with 22 min for the anterior lateral approach, 35 min for the upper lateral approach and 42 min for the posterior lateral approach. Regarding the surgical complexity of the surgery, three cases of the low window approach were rated as “easy” surgery, whereas one case was rated as “moderate” surgery. For the anterior-lateral window approach (CLA), two cases were rated as “difficult”, and one was rated as “moderate” surgery due to difficulty differentiating between the nasal cavity and maxillary sinus. One surgical access in the posterior-lateral window approach was rated as highly difficult due to profound bleeding and difficulty locating the displaced implant. Difficult surgeries were encountered when handling the upper-lateral window due to the thickness of the zygomatic bone (Table 2).

**Table 2:** Summery of the surgical approaches and the outcome of the different techniques.

Variable	Surgical access	Time required to remove implant (min)	Surgical complexity	Pain^a^	Foul taste	Post nasal drip	Facial swelling	Nasal obstruction	Facial paresthesia
Patient 1	LW	10	Easy	Mild	Insignificant	Reported	Mild	Mild	Not detected
Patient 2	LW	15	Moderate	Mild	Insignificant	Not reporter	Mild	Mild	Not detected
Patient 3	LW	8	Easy	Mild	Insignificant	Not reporter	Moderate	Mild	Not detected
Patient 4	LW	13	Easy	Mild	Insignificant	Not reporter	Moderate	Mild	Not detected
Patient 5	ALW	25	Moderate	Mild	Significant	Reported	Moderate	Mild	Not detected
Patient 6	ALW	20	Difficult	Moderate	Significant	Reported	Moderate	Moderate	First 4 weeks then resolved
Patient 7	ALW	22	Difficult	Mild	Significant	Reported	Moderate	Moderate	First 7 weeks then resolved
Patient 8	ULW	35	Difficult	Moderate	Highly significant	Reported	Sever	Sever	First 9 weeks then resolved
Patient 9	ULW	35	Difficult	Moderate	Significant	Reported	Moderate	Moderate	Not detected
Patient 10	PLW	40	Highly difficult	Moderate	Significant	Reported	Moderate	Moderate	Not detected
Patient 11	PLW	44	Difficult	Moderate	Significant	Reported	Moderate	Mild	Not detected

*LW* lower window, *ALW* anterior-lateral window, *ULW* upper-lateral window, *PLW* posterior-lateral window.

^a^Average pain scores during the first 7 days after the surgery.

The average pain from the day of the surgery until 7 days post-operative was “mild” in all cases with the lower window approach. In the anterior lateral window, the average pain was mild in 2 cases and moderate in only 1 case. All pain was reported as “moderate” following implant removal from the upper lateral and posterior lateral approaches. Regarding neural injury, three patients developed temporary paraesthesia, of which two patients had paraesthesia in the buccal vestibule following the anterior-lateral window approach near the canine fossa with a full recovery time ranging from 4 to 7 months. The recovery time in the third case extended to week 9, which was related to surgery in the upper lateral approach in which numbness at the site of the nose, upper lip and skin over the zygomatic bone was reported.

Nasal obstruction in 6 patients was scored on post-operative day 7 as mild and reported as moderate in 4 patients, which resolved by post-operative day 14. In one patient, the nasal obstruction was severe, and the patient also suffered a highly significant foul taste with a post-nasal drip in the mouth and significant facial swelling. In this case, the upper lateral approach was followed to remove the displaced implant, and the patient reported full resolution of the nasal obstruction and post-nasal drip after 4 weeks. The foul taste was insignificant in the low window approach cases; for the same approach, detectable post-nasal drip was reported only in one case. Both upper lateral and posterior lateral approaches were associated with a significant foul taste. Post-nasal drip was reported in 8 cases out of 11 and was resolved within the first 4 weeks after surgery. Facial swelling following surgery was severe in one case in which the swelling extended to the lower eyelid and caused partial eye closure, moderate in eight cases and mild in two cases; all cases were resolved completely 10–14 days post-operatively.

Three months after the surgery, all the patients reported sinus-free symptoms. OPG X-rays showed no evidence of any sinus fluid or cyst. Digital palpation of the bone area where the surgery was performed showed clinically detectable depression in the area of the CLA in two patients. Moderate horizontal and vertical alveolar bone resorption and shrinkage were associated with the lower window approach (three cases). Vestibular shortening and visible scarring were detected in one patient that underwent the upper lateral approach, but the patient was not troubled.

## Discussion

Migration of dental implants into the maxillary sinus is considered iatrogenic because it mostly occurs due to a lack of proper surgical procedure planning. Varol et al.^10^ and Chappuis et al.^11^ listed some causes of displacement during surgery, including less experienced surgeons, poor primary implant stability, unsuccessful bone regeneration following previous maxillary sinus floor elevation, and implantation without treating perforation caused by implant drilling. In particular, poor primary stability causes implant micromovement, which prevents clot formation and revascularization and makes new bone formation more difficult. All of these factors can lead to poor implant fixation and failure to achieve osseointegration, resulting in late implant displacement^12–14^.

Consequently, an implant displaced in the maxillary sinus often results in serious complications such as maxillary sinusitis, nasal obstruction, bony necrosis, foreign body aspiration, and migration into deeper sinus cavities. Therefore, a migrated implant in the maxillary sinus should be removed as early as possible to prevent further risks of worsening symptoms and uneventful sequelae^15–17^. The Caldwell Luc surgical approach has been one of the most favourable classical approaches to the maxillary sinus due to its ease of access and visibility. However, several post-operative complications have been reported following CLA, such as post-operative maxillary cysts (POMCs) and a high rate of relapse regarding sinus symptoms, which were thought to be induced by decreased sinus volume resulting from inferior osteotomy^18^.

On the other hand, the intracrestal approach is recommended by many clinicians due to its direct access, rapid recovery of sinus functions, low invasiveness, and flexibility. The crestal approach requires less bone volume removal and less surgical trauma, as the bone is less dense and more trabecular than the lateral wall of the maxillary sinus. However, this blind procedure may lead to unsatisfactory results when the material is entrapped in the undercut of the sinus and often leads to undesirable post-operative depression of the alveolar ridge due to the procedure required to enlarge the socket for a suction tube^19,20^.

In the current study, the intracrestal approach was associated with less pain and less facial swelling than the CLA. The approach was distant from the infraorbital nerve, the main sensory nerve in the labial gingiva and upper lip, and there were no neuropathies reported in the present study following the intracrestal approach. The maximum time required to remove the implants through this approach was 13 min compared to 44 min in the posterior lateral approach, which is attributed to the nature of the alveolar bone as it can be easily removed to gain direct mechanical and visual access to the sinus. Less surgical manipulation resulted in less pain, as all 4 patients that underwent this approach presented with mild pain in the week following the surgery.

On the other hand, the Caldwell-Luc approach is the gold standard for obtaining access to the maxillary sinus for the treatment of various problems, including retrieval of foreign bodies. There are some disadvantages encountered in this approach reported in the current research, including the resulting bone defect of the lateral antral wall and injury of the mucosal branches of the infraorbital nerve. Our findings related to the complications related to CLA were in accord with previous studies^21–24^. Chiapasco et al.^18^ retrospectively analysed paranasal sinus complications following displacement of oral implants in the maxillary sinus treated according to the patients’ clinical situation by functional endoscopic sinus surgery (FESS), an intraoral approach, or a combination of both procedures. The main limitation of the CLA surgical procedure is partial or no recovery of maxillary sinus functions related to the patency of the maxillary ostium.

A more serious nerve injury was reported in one case associated with the upper lateral approach, and facial paraesthesia involved both the oral mucosa and the skin on the lateral side of the nose, which resolved 9 weeks after the surgery. The upper lateral antrostomy was carried out at the premolar-molar region, and the flap extended more superiorly to expose and remove the thick zygomatic bone using a round bur, which might be the possible cause of injury to the infraorbital nerve. The time required to remove the implant was 35 min, and the surgery was scored as difficult due to challenge of removing the bone and the pain was reported as moderate; in one case, the swelling was severe.

Locating an implant inside the sinus is extremely difficult due to the large size of the sinus. The average capacity of the maxillary sinus varies from 9.5 to 20 mL and averages 14.75 mL. The average dimensions were 3.75 cm vertically, 2.5 cm mediolaterally and 2.5 cm antero-posteriorly. The sinus cavity may also extend into the zygoma. Thus, a sinus can usually be filled with 10–20 mL of lavage saline^25,26^. Considering the position of the implant in the maxillary sinus, it is often difficult to visualize the implant. In particular, visualization is difficult when the implant is located on the superior or posterior regions. It is usually a challenge to remove through the intracrestal or Caldwell Luc approach.

Posterior displacement of the implant is a real challenge due to accessibility. Dryer and Conrad^27^ reported a case regarding surgical complications related to implants in the pterygomaxillary region. In their report, the dental implant was removed with a CT-guided endoscope transnasally. In the current study, two of the implants displaced into the posterior aspect of the maxillary sinus were retrieved by the posterior lateral window approach. The surgery took 42 min on average despite the soft bone that existed in this area, which was removed easily. The longer time taken to remove the implant was attributed to bleeding from the pterygoid venous plexus. Another challenge in this approach was the difficulty of locating the implant as it changed its position according to the patient’s position. It is worth knowing that this only occurred in the only case in which the surgery was performed within the first 24 h of displacement.

Immediate or early removal of the displaced implants has been suggested to prevent infectious complications due to contact of the implant with the mucosa of the sinus interior^28^. However, immediate displacement of the implant deep inside the sinus after sinus lifting with unnoticeable membrane perforation renders the implant free and allows it to change positions due to the nonattachment status of the implant to the sinus membrane. Thus, the authors recommend leaving the implant in situ for 2 weeks to allow granulation tissue encapsulation.

In 2000, Lida et al.^29^ reported a case of a patient who underwent dental implant installation to replace a second upper molar. Five years later, the patient noticed mobility of the implant: the prosthesis was removed from the implant, but the implant was left in position. Eleven years later, a panoramic radiograph revealed displacement of the implant into the right maxillary sinus, and the implant was removed by an intraoral approach under local anaesthesia. The patient recovered uneventfully with no evidence of sinus infection. Raghoebar and Vissink^30^ reported a case of a man who underwent three implant installations. After three months, the migration of an implant into the maxillary sinus was discovered after a panoramic radiograph. The implant was removed in association with a sinus graft under general anaesthesia through the lateral wall of the maxilla.

Kitamura^31^ reported a case of a woman with discomfort in the right maxilla and a discharge of pus from the nose. Panoramic radiographs and computed tomograms showed the presence of an implant in the right maxillary sinus. The patient was treated under general anaesthesia using a rigid endoscope 4 mm in diameter equipped with a digital video unit. The middle nasal meatus and the semilunar hiatus were visualized; the implant was placed in the posterior portion of the middle nasal meatus and was removed from the maxillary sinus with forceps. The patient was discharged from the hospital one week later and had no further trouble. Kluppel et al*.*^32^ reported two cases of dental implants displaced in the maxillary sinus. One of them was removed and sinus lifting was performed. The procedure was performed under local anaesthesia using a lateral antrostomy approach, and the sinus membrane was elevated and incised to allow removal of the dental implant; the other one was kept in place with no complications after a 5-year follow-up.

This study has some limitations. First, the sample size was too small to establish the effectiveness of the single best approach. Second, the range of maxillary sinus status between the different groups was not the same. Although further study of a large population is needed to establish definite indications and limitations of each approach, our study provides a comparison between the four approaches, showing that surgical access is comparable.

## Conclusion

The choice of the most appropriate surgical approach to retrieve a displaced dental implant in the maxillary sinus depends on the location of the implant inside the sinus. The most straightforward approach is through the implant site, and this approach has fewer surgical complications than other approaches. To confirm the efficacy of different intraoral approaches, a larger cohort with a long-term follow-up study is recommended.

## Data Availability

The authors declare that all the data supporting the findings of this study are available within the article.
